# The practice of public health coordination in Sweden: roles, responsibilities, and realities

**DOI:** 10.1093/heapro/daaf163

**Published:** 2025-09-29

**Authors:** Isa Norvell Gustavsson, Frida Jonsson

**Affiliations:** Department of Epidemiology and Global Health, Umeå University, 901 87, Umeå, Sweden; Department of Epidemiology and Global Health, Umeå University, 901 87, Umeå, Sweden

**Keywords:** public health policy, public health practice, coordination, intersectoral collaboration, health promotion, Sweden

## Abstract

In response to increasingly complex and cross-sectoral public health challenges, coordination has emerged as a key strategy for aligning efforts across fragmented systems. However, despite its widespread endorsement, coordination remains conceptually ambiguous and difficult to operationalise in practice. This qualitative study explores how public health coordination is enacted at the local level in Sweden, where municipalities employ public health coordinators to promote population health and reduce inequalities. Semi-structured interviews with 21 public health coordinators across diverse Swedish municipalities were conducted and through an inductive thematic analysis four key themes were developed: driving targeted efforts and holding processes together; connecting activities to policy goals through purposeful planning; creating conditions for collaboration by engaging relevant stakeholders; and building a knowledge support function through acquiring and sharing new knowledge. The findings reveal that coordination is a dynamic and adaptive function requiring strategic thinking, relational skills, and contextual sensitivity. Effective coordination depends not only on individual competencies, such as communicative, diplomatic, and administrative abilities but also on structural conditions, including political mandates, formalised goals, and sufficient time and resources. Coordinators often operate without formal authority, relying on trust and leadership support to navigate complex and shifting responsibilities. The study concludes that coordination is essential for enabling collaboration, sustaining public health efforts, and aligning activities with policy goals. It highlights the need for clearer role definitions, supportive frameworks, and further research into how coordination contributes to long-term public health outcomes across different domains and local contexts.

Contribution to Health Promotion

- Coordination is widely promoted as a proper response to complex public health challenges but is often vaguely defined and hard to implement.
- The findings emphasizes that coordination is essential for keeping public health issues visible and ensuring long-term impact.
- The study identifies key conditions, such as time, support, and clear mandates, which help coordinators work effectively.

## INTRODUCTION

In an era marked by climate change, population ageing, persisting inequalities, and the ‘invisible epidemic’ of non-communicable diseases, compounded by acute crises such as the COVID-19 pandemic, the field of public health faces numerous interconnected and complex challenges, so-called ‘wicked problems’ (Rittel and Webber 1973, p. 160, Weber and Khademian 2008, Conradie and Boshuijzen-van Burken 2020). These challenges are further shaped by the commercial determinants of health, as corporate practices and market-driven influences often exacerbate health inequities and complicate police responses (Baum *et al*. 2023, Gilmore *et al*. 2023). The wickedness of these problems lies in their unstable and emergent nature, where both causes and effects are often multiple, unforeseen, and context dependent. This complexity makes them difficult to define and even harder to solve (Weber and Khademian 2008). As Rittel and Webber (1973, p. 160) famously noted, wicked problems are, at best, ‘resolved over and over again.’ These challenges are further complicated by their cross-cutting nature, as they typically transcend policy areas, organizational structures, and professional boundaries (Axelsson and Axelsson 2006, Weber and Khademian 2008). As such, within fragmented and siloed institutional landscapes, addressing such challenges often requires multi-actor and intersectoral approaches, which in turn demand effective coordination to be successful (Axelsson and Axelsson 2006, Hunter and Perkins 2012, Duit and Löf 2015).

There is a widespread trust in coordination as a proper response to complex public health problems that involve or necessitate multiple actors and organizations. However, coordinating public health practice is itself difficult and complex (Hunter and Perkins 2012, Ekholm and Svensson 2020). Despite this, it is often viewed as a ‘magical concept’ (Pollitt and Hupe 2011, p. 643) that appeals to a broad audience and promises universal solutions to complex problems (Pollitt and Hupe 2011). Like other popular organizational concepts such as innovation, trust, and governance (Bentzen 2019), coordination is often vaguely defined and subject to conflicting interpretations. This ambiguity increases the risk that the concept becomes diluted, holding primarily symbolic value, that is easy to endorse, but difficult to operationalize (Bentzen and Bringselius 2023). This tension is reflected in the literature, where coordination is often presented as a trusted solution to complex public health challenges, yet, rarely unpacked in terms of what it entails or how it is operationalized in practice.

Despite the potential benefits of coordination, not all coordination effort succeeds in practice (Hunter and Perkins 2012). To gain a deeper understanding of coordination, there is a need for empirical insights into how coordination is enacted and what such practices require. In this study, we share experiences from Sweden, where, similar to Norway (Hagen *et al*. 2018), public health coordinators have been working at local and regional levels to promote population health and reduce health inequalities. While similar roles, such as *health brokers* (van Rinsum *et al*. 2017) or *knowledge brokers* (Traynor *et al*. 2014), are discussed in the international literature, the broad and strategic use of public health coordinators appear to be relatively unique to the Nordic context. By exploring how these coordinators work and what their roles entail, the current study offers valuable insight into how public health can be systematically strengthened through coordinated efforts at the local level.

Before presenting findings from a qualitative study involving interviews with 21 public health coordinators across Swedish municipalities, we first situate the coordinator role within the broader international literature. We also describe how public health policy and practice are organized in Sweden to provide context for our analysis.

### Coordination, and its connection to related concepts

Despite its recognized importance in public discourse and policy, the term *coordination* is often used interchangeably with *cooperation* and *collaboration*, making conceptual clarity essential (Axelsson and Axelsson 2006). According to the Swedish National Board of Health and Welfare (2005, 2025), *coordination* involves aligning efforts, recourses, and activities based on shared rules and objectives to improve quality and efficiency in multi-actor contexts. In contrast, *cooperation* refers limited, short-term joint activities, while *collaboration* entails broader, organizational level engagement for a specific purpose (Swedish National Board of Health and Welfare 2005, 2025). Jacobsson (2011) argues that *collaboration* differs from *coordination* in that it emphasizes working together in a general sense, rather than achieving concrete, collective outcomes.

Jacobsson (2011) further suggests that the relationship between these concepts is primarily a matter of conceptual precision. *Collaboration* is the least precise, focusing broadly on ‘working together’, while *coordination* is the most precise, emphasizing ‘acting together’ to achieve specific goals. Similarly, Hjortsjö (2006) distinguishes coordination as the process of organizing joint efforts to achieve results, while collaboration and cooperation involve interaction and integration between. Hjortsjö (2006) also distinguishes between *formal collaboration*, structured and goal-oriented, and *informal collaboration*, which arises from participants’ willingness to cooperate.

**Figure 1.**
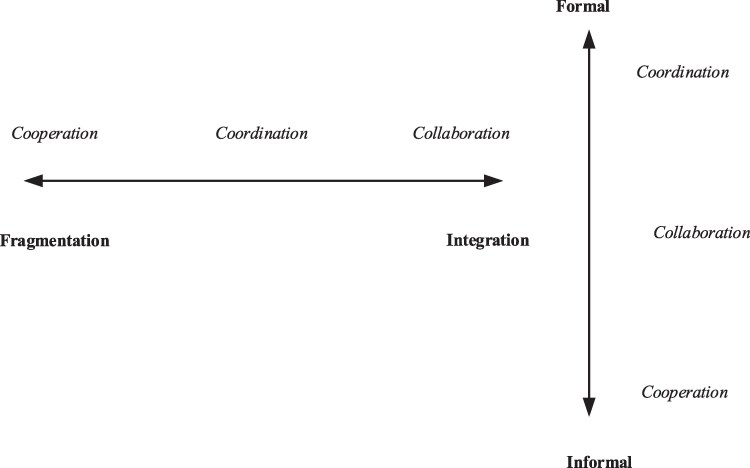
Overview of the relationship between coordination, cooperation, and collaboration according to a horizontal (integration) and vertical (formality) perspective.

In the international organizational literature, these concepts are often places on a continuum ranging from *fragmentation*, where organizations operate independently and autonomously, to *integration*, where organizations are interconnected and interdependent (Keast *et al*. 2007, McNamara 2012). As illustrated in Fig. 1, *cooperation* lies closest to fragmentation, characterized by voluntary, interest-driven interactions. *Coordination* occupies the middle, involving structured efforts towards shared goals (Keast *et al*. 2007, McNamara 2012). *Collaboration,* closest to integration, is defined by interdependent tasks and mutual responsibility.

Viewing these concepts along a horizontal continuum emphasizes the degree of integration among actors, particularly in terms of knowledge exchange, power sharing, and interdependence (Keast *et al*. 2007). To complement this, McNamara (2012) introduces a vertical dimension that considers the level of *formalisation*. On this axis, *cooperation* is the most informal, often developing spontaneously when actors recognize mutual benefits in sharing information or resources. *Coordination,* by contrast, is the most formal, typically initiated through hierarchical structures and formal agreements such as contracts or joint action plans. *Collaboration* falls between these two extremes on the vertical axis. It often arises through a blend of formal and informal mechanisms, while formal agreements may initiate the collaboration, the ongoing work is often sustained through personal relationships and informal networks rather than contractual obligations.

While the literature offers a broad and generic conceptual understanding of coordination, two distinct perspectives emerge on how the concept can be interpreted. First, coordination is frequently understood as an organizational, process-oriented action (i.e. to *coordinate*) through which multiple organizations or professional groups share risks, tasks, information, and knowledge to achieve common goals (Keast and Mandell 2014). Alternatively, coordination can be viewed as a person-based function (i.e. being a *coordinator*) performed by individuals who bring actors together in meaningful and sustained collaboration. From this perspective, coordination becomes a more permanent and clearly defined role and responsibility.

Regardless of whether it is seen as an action or a function, coordination is often described in abstract and conceptual terms (Keast *et al*. 2007, McNamara 2012, Keast and Mandell 2014), making it difficult to understand how it translates into practice. Although empirical insights into the practical nature of coordination are limited, it is generally assumed to improve the quality and efficiency of public health efforts by combining resources (Axelsson and Axelsson 2006, Koelen *et al*. 2008, Hunter and Perkins 2012) and minimizing overlaps and duplication (Dötterweich 2006, Hämäläinen *et al*. 2016, Weiss *et al*. 2016). However, despite its potential benefits, coordination does not always succeed in practice (Hunter and Perkins 2012). There remains a limited understanding of what constitutes effective coordination and what conditions are necessary for it to function well.

Against this backdrop, this study adopts a perspective of coordination as a person-based function, with the aim of exploring how public health coordinators in Sweden work and what their role entails in practice.

### Public health policy and practice in Sweden

Sweden’s public health policy is guided by a national framework consisting of one overarching goal and eight target areas (The Swedish Public Health Agency 2024). The overall goal emphasizes equitable health, aiming to create societal conditions that promote good and equitable health for the entire population and to close avoidable health gaps within one generation.

The Swedish Public Health Agency is responsible for coordinating and monitoring public health efforts across governmental authorities, regions, and municipalities. Given that public health is a cross-sectoral field—spanning multiple sectors and organizations—the agency underscores the importance of coordination, while acknowledging the challenges posed by the complexity of involving diverse stakeholders (The Swedish Public Health Agency 2020). Due to Sweden’s decentralised governance structure, significant responsibility for public health lies with the country’s 21 regions and 290 municipalities (The Swedish Public Health Agency 2024), each of which has considerable autonomy and independent taxation powers. While the regions are administratively responsible for healthcare, the municipalities oversee areas such as child and elderly care, education, social services, and community planning. This allows them to tailor their public health efforts to local needs and conditions (The Swedish Public Health Agency 2024).

This study focuses on the municipal level, specifically on the role of public health coordinators, who are often employed to initiate, support, and sustain public health activities at local levels. These coordinators are tasked with integrating public health perspectives into governance and management processes, monitoring and analysing public health trends, identifying and prioritizing needs, and leading public health initiatives in collaboration with various stakeholders. Their work is expected to be systematic and knowledge-based (The Swedish Public Health Agency 2024). However, there is considerable variation across municipalities in how the coordinator role is structured. Differences exist in terms of organizational placement, role definition, and the degree of coordination responsibility. While some coordinators work full-time in this capacity, others combine coordination with additional responsibilities (The Swedish Public Health Agency 2024).

## MATERIALS AND METHODS

This qualitative study involved semi-structured interviews with 21 coordinators working in the field of public health across municipalities of varying sizes and geographical location in Sweden. To support clarity and ensure accurate interpretation of the findings, the study incorporated participant involvement beyond data collection. In line with recommendations for active engagement throughout the research process (Braun and Clarke 2021, George *et al*. 2023), the coordinators were invited to a digital meeting to comment and discuss preliminary findings.

### Recruitment and participants

Coordinators with at least 1 year of experience working in the role were recruited based on a stepwise procedure. From a list of 40 public health coordinators compiled by the Swedish Public Health Agency that were part of their national networks, we contacted each one via e-mail to present the study and its purpose while asking if they would like to participate in an interview. Our request was not answered by 12 coordinators, while 28 expressed an initial interest in participating. Of these, 7 declined for various reasons and the remaining 21 accepted and were included in the study.

Several of the participants had a coordinating responsibility in several specific area of public health focusing, for example, on substance abuse, mental health, and/or suicide prevention but also crime prevention, gender-based violence, equity, and social sustainability. Most of the participants had a permanent position and were employed by the municipality where between 5% and 100% of their time were dedicated to the coordination role. Their experience of working as public health coordinators ranged from over 20 years to 1 year (Median 4).

### Data collection and analysis

Data were collected through individual interviews with the 21 coordinators in September and October 2023. Informed by the study’s aim, the authors collaboratively developed a semi-structured interview guide. This format allowed for flexibility in exploring emergent topics during the interviews, while ensuring coverage of predetermined areas of interest. The guide included questions about the coordinators’ roles, the rationales, and goals underpinning their work and perceived opportunities and obstacles in public health coordination. A pilot interview was conducted with one coordinator to assess the clarity and relevance of the guide. As the pilot yielded rich and detailed responses, no revisions were deemed necessary, and the interview was subsequently included in the dataset. All interviews were conducted through video conference, ranged between 31 and 57 minutes and were recorded by a digital audio recorder with participants’ permission. The interviews were transcribed verbatim leaving out personal identifiers. In conjunction with the interviews, participants answered a short online questionnaire with some background information about their education, work experience, and area of responsibility as detailed above (Table 1). While most answered the questionnaire prior to the interview, a few answered after the interview was conducted and two participants did not complete the questionnaire.

**Table 1.** Participant demographic (*N* = 19, questionnaire data lacking from *N* = 2).

	Category	Number (N)
**Area of responsibility**	Substance abuse	5
	Substance abuse, mental health and suicide prevention	3
	Mental health	1
	Mental health and suicide prevention	9
	Suicide prevention	1
**Percentage of coordination responsibility**	5%–49%	4
	50%–100%	15
**Years of coordination experience**	1–3 years	10
	4–9 years	6
	+10 years	3

In January 2024, when preliminary themes had been developed during the initial reflexive thematic analysis, all participants were invited to a digital meeting. The meeting, which served as a form of member-checking (Shenton 2004), had a two-fold purpose of (i) presenting findings to participants and (ii) discussing the findings to validate and refine our interpretations. The discussion was transcribed in real time, and the transcripts were then anonymised and lightly edited for readability before being integrated into the analysis. Material from interviews and the digital meeting was analysed using inductive thematic analysis (Braun and Clarke 2021). Based on the two-phased data collection, the analysis process was also conducted in two phases. The first phase started with a thorough reading of the interview transcripts, focusing on identifying recurring patterns. The transcripts were then coded and grouped together to construct themes. The dialog meeting transcripts were then integrated in the analysis, which refined the themes both relating to content and definition.

### Ethical considerations

All participants received written and oral information regarding the study and its purpose, that participation was voluntary, and they were within their rights to cancel their participation at any time without giving a reason or be subjected to negative consequences. They all gave their written consent to participate in the study. No sensitive personal data were handled in this study, which means that ethics approval is not required according to the Swedish Ethical Review Act. Data handling and storage follows the General Data Protection Regulation and other Swedish legislation, following Umeå University procedures.

## RESULTS

Through an inductive thematic analysis, four themes were developed, which provide insights into how public health coordinators in Sweden work and what their role entails in practice. In the themes, we outline the role of the public health coordinators, detailing the role’s strong connection to the understanding of coordination as a function, through which coordinators create opportunities and conditions for different organizations to achieve common goals in public health work. The themes depict how coordination operates and is operationalized in practice by ‘driving targeted efforts, planning and implementing purposeful activities, engaging relevant stakeholders, and acquiring and sharing knowledge’.

### Driving targeted efforts and holding processes together

This theme illustrates how public health coordination at the local level is fundamentally about holding processes together in pursuit of policy goals set by elected municipal politicians. Coordinators described their work as an ongoing effort to steer public health initiatives in a direction that supports positive development within the municipality. Many coordinators likened their role to being the ‘spider in the web’, describing coordination as the glue that holds together diverse processes. These processes spanned both strategic and operational levels as Coordinator A (substance abuse) described:

This week, I am developing strategical documents and mind maps on how various policy documents should be interconnected and presenting them to the highest political level/…/To then be distributing start numbers at an event, to raise awareness about mental health and the importance of physical activity for well-being.

Given the complexity of local public health practice, which often involves diverse activities and actors, a cohesive and guiding function was perceived as essential to ensure progress. Interviews revealed that coordination requires a certain degree of formalization, guided by objectives set by municipal politicians. The importance of political anchoring and governance was strongly emphasized by the coordinators. These political decisions were seen as crucial not only for clarifying what should be prioritized, but also for legitimizing which activities, actors, and approaches should be coordinated. As Coordinator B (mental health and suicide prevention) explained:

Decisions, decisions, decisions. That is, political control like a political club. I can say… Well, suicide, the work currently being done on suicide prevention. I don’t think I would have taken it on if there hadn’t been a political decision about it… We couldn’t have gone to those managers and said, ‘Hey, 400 employees need to be trained in Mental Health First Aid’.

At the same time, coordinators noted that public health objectives are often subject to change due to a steady flow of updated guidelines, emerging needs, and shifting political priorities. This required coordinators to adapt quickly, balancing structure and flexibility in their work. Coordination often involved tasks that spanned sectoral, organizational, and professional boundaries, requiring a degree of structure to manage complexity. While some coordinators had clearly defined strategic roles, others moved more fluidly between management and frontline work. Several appreciated this alternating nature of the role, describing it as a key reason they enjoyed their work. However, the broad and sometimes fragmented scope of their responsibilities also posed challenges, as they were often expected to be ‘everywhere at once’ in driving targeted efforts and holding processes together towards achieving established policy goals.

### Connecting activities to policy goals through purposeful planning and implementation

This theme highlights that public health coordination at the local level is not only about holding processes together, but it also involves the purposeful planning and implementation of activities to achieve goals outlined by policy. Coordinators described how their work encompassed a wide range of activities aligned with the public health objectives outlined in local policies. At the strategic level, this included leading networks of managers, developing policy documents, and mapping existing efforts within the municipality. At the operational level, activities ranged from organizing training initiatives and conferences to engaging directly with citizens.

For those whose roles spanned both strategic and operational levels, planning could involve everything from ordering coffee and booking venues to designing and delivering the activities themselves or supporting others in doing so. While some appreciated this variety, others found the dual nature of the role challenging. The constant shifting between levels could fragment their work, making it difficult to follow up on commitments or maintain focus. As Coordinator H (Suicide prevention) put it ‘*I stretch myself rather thin*’. Regardless of the level or form of activity, participants emphasized the importance of purposeful alignment with policy goals:

There has to be a/…/a purpose when we summon to training initiatives or conferences or choosing a focus area. And that we have a purpose of meeting and a purpose with the training in certain programs. I find this extremely important. It is my mantra. If you don’t know why you are doing what you are doing, you should probably do something different. If you can’t even answer ‘why am I doing this?’ (Coordinator C, mental health)

Coordinators stressed that activities needed to be goal-oriented, clearly linked to municipal targets. These connections not only legitimised the activities but also helped clarify roles and responsibilities for involved actors, many of whom struggled to see how public health goals related to their core or statutory operations. Purposefulness also involved prioritization. Given the breadth and complexity of public health issues, coordinators often faced expanding responsibilities. To prevent unrelated tasks from diverting resources and focus, they had to constantly balance and prioritize. However, setting boundaries was not always straightforward, and the order of priorities was sometimes unclear.

### Creating conditions for collaboration by engaging relevant stakeholders in joint action

This theme illustrates the complex nature of coordinating public health practices involving different organizations or professions. Coordinators emphasized the complexity of the public health issues they were tasked with coordinating, where no single organization or profession alone could shoulder the responsibility, stating that these areas demand collaborative efforts that cut across organizational and professional boundaries. Against this backdrop, coordinators emphasized that coordination largely involved creating the conditions for joint action by actively involving relevant stakeholders. As Coordinator D (substance abuse) explained:

I believe that it is bringing stakeholders together, to structure, and direct the work. Because the work I’m involved in include various stakeholders and is often cross-sectoral, both within the municipality and externally with authorities and civil society in a rather diverse crowd, I should try to guide and synchronize our forces. To favour some kind of common purpose or agreed-upon goal, that is my thought.

Many stakeholders were described as unaware of their roles or responsibilities in the shared work of public health. Coordinators often had to inform, engage, and motivate them to participate. Without a designated coordinator, the coordinators feared that public health issues could easily be deprioritized in favor of more immediate or core operational concerns.

If you don’t have anyone specifically appointed—you are coordinator for this issue—it can easily be down prioritised since the focus is on the main operations/…/Yes, well then, that issue might disappear because the focus is on the main operations. (Coordinator E, mental health and suicide prevention)

To successfully engage stakeholders, coordinators had to consider the varying conditions across organizations, including competing demands for time and resources. In this context, the ability to approach stakeholders with responsiveness, humility, and curiosity help coordinators gain insight into the challenges stakeholders faced and fostered mutual understanding.

Interestingly, the challenge was not in adopting a humble or responsive demeanor, this came naturally to most coordinators, but rather in navigating the complexity of the issues themselves. Stakeholders across health and social sectors often struggled to understand why they should be involved in public health efforts. As a result, coordinators frequently had to clarify the purpose and relevance of joint efforts in areas such as substance abuse, mental health, and suicide prevention, emphasising that successful collaboration depends on the contributions of multiple actors.

### Being a support function by acquiring and sharing new knowledge

This theme illustrates how public health coordination at the local level involves not only organizing and facilitating collaboration, but also acquiring and sharing knowledge, both within the municipal organizations and across national, regional, and local networks.

Coordinators emphasized the importance of gathering information and evidenced based knowledge from sources such as guidelines, reports, and scientific literature, and disseminating it to relevant actors within the municipality. The goal was to create a shared knowledge base to support various public health efforts. To avoid overwhelming colleagues with information, they described the need to process and ‘package’ knowledge in a way that made it accessible and relevant. In this regard, coordination served as a procedural and knowledge support function, ensuring that the right information reached the right people. This required careful judgment about who needed what information, and how much could be shared without it being lost in the broader information flow.

Acquiring and disseminating knowledge is also an important part of coordination, to find some way of ensuring that everyone rests on the same knowledge base and has an understanding of how our situation looks, or what challenges we face, or what prerequisites there are. I believe that is also a part of my job as a coordinator: to ensure that everyone has the right knowledge. (Coordinator F, substance abuse, mental health, and suicide prevention)

The ability to assess and disseminate relevant knowledge was supported by a basic scientific understanding, including the ability to evaluate the quality of research and interpret scientific findings. Coordinators with a background in public health or experience in reading academic literature appeared better equipped to identify and share credible information. However, this work also required time to stay updated with emerging research. Coordinators with more strategic roles reported having greater opportunity to engage in this work, while those who frequently shifted between strategic and operational tasks often struggled to find the time.

External support from national and regional stakeholders was seen as valuable, as it provided access to information that coordinators might not have the capacity to gather themselves. Still, even when information was provided, it required time and effort to review, adapt, and disseminate it effectively. Coordinators emphasised that they could not rely solely on others for knowledge, they needed the resources and autonomy to seek out relevant information independently. In addition to formal sources, peer networks, especially with other public health coordinators, were described as vital for knowledge exchange. As Coordinator G (substance abuse) noted:

For us to be able to implement good activities and initiatives in our municipality, we need to receive knowledge. And we are dependent on… well, for one, meeting organisations and such. Second, to collect a lot of data for us so that we have as good knowledge as possible/…/So, we are dependent on getting the support and input from… well, national actors, regional actors, but mainly perhaps from local actors.

While many coordinators were active in sharing knowledge with others, some expressed frustration at the lack of reciprocity, particularly from local stakeholders, but also at times from regional and national levels. Beyond knowledge sharing, these networks also served as collegial support systems, offering opportunities to consult, brainstorm, and exchange ideas. Coordinators described these informal exchanges as essential for avoiding duplication of effort, ‘not having to reinvent the wheel’, and as a source of inspiration for trying new approaches. However, in many cases, formalised forums for such exchange were lacking, and it was often up to individual coordinators to initiate and maintain contact with peers in similar roles.

## DISCUSSION

The findings from this study underscore that coordination in public health is not merely a logistical challenge, but rather a response to the wicked nature of the problems it addresses. Wicked problems are complex, lack clear solutions, and involve multiple stakeholders with conflicting values and priorities (Rittel and Webber 1973, Weber and Khademian 2008). Coordinators must navigate diverse perspectives, operate across organizational boundaries, and adapt to shifting contexts, hallmarks of wickedness in public health issues. Role ambiguity, informal authority, and the need to work at both strategic and operational levels further illustrate the dynamic terrain that coordinators must manage. In this light, coordination is not a technical fix, but a strategic and relational practice that builds collective capacity to address evolving challenges. The findings show that effective coordination takes on different forms. It is not only about *what* coordinators do (i.e. activities), but also about *how* they do it (i.e. their skills and attitudes).

Coordinators primarily focus on creating conditions for collaboration across organizational and professional boundaries. This requires the ability to identify and engage relevant stakeholders. Given the diversity of backgrounds and perspectives, coordinators must be able to listen attentively to stakeholders’ needs and concerns, while also articulating the benefits of working together. Previous research has primarily focused on the skills and attitudes of coordinators when discussing the coordination role. In line with this, the findings from this study suggest that effective coordination in public health is supported by a combination of communicative, diplomatic, and administrative abilities. While administrative skills help coordinators navigate organizational culture and manage potential conflict (Axelsson and Axelsson 2006, Dötterweich 2006, Hunter and Perkins 2012, Andersson *et al*. 2019), communicative and diplomatic abilities foster knowledge exchange, motivate stakeholders, and build trust-based relationships (McGuire 2006, Nordqvist *et al*. 2009, de Jong *et al*. 2023). As Dötterweich (2006) notes, coordinators must be patient, determined, and flexible to manage the broad and evolving nature of their role, qualities that were also evident among coordinators in this study.

Less discussed in the literature, however, are the conditions and resources coordinators need to carry out their work effectively. While financial security has been highlighted as a key factor (van Rinsum *et al*. 2017), the findings here underscore the importance of time, space, and support. Coordinators need time not only to plan and implement activities but also to engage stakeholders, monitor developments, and stay informed about the state of knowledge and the internal workings of the municipality. Further, coordinators need support in prioritizing and setting boundaries, as well as receiving encouragement and recognition of their work. At a strategic level, clear directives, such as action plans, mission statements, and formal agreements are essential. These frameworks provide both direction and structure, helping coordinators avoid the risk of their work becoming vague or limitless. Without such frameworks, coordination is hindered, and a lack of formal backing makes it harder to motivate activities or engage other actors.

According to van Rinsum *et al.* (2017), coordinators should operate at both strategic and operational levels, as this dual positioning enables more comprehensive and impactful work. However, this also adds to the complexity of the role. The wide range of tasks can make it difficult to maintain an overview, and the role itself may be perceived as diffuse, unclear, or even invisible to others. These ambiguities, especially around role definition, were also evident in this study and are seen as barriers to coordination. Coordination in public health practice is also characterized by the need to balance flexibility and structure. The work is often highly delimited, shaped by shifting priorities and boundaries. As Hämälainen *et al.* (2016) notes, coordination is both complex and contextual, requiring approaches and methods to be adapted to prevailing circumstances to foster cohesion among stakeholders. Since coordinators often lead without formal managerial positions, they rely heavily on support, trust, and a clear mandate from leadership. This support is essential not only for legitimising the issues and approaches they promote, but also for helping them set priorities and define boundaries (McGuire 2006). Without clear political frameworks and structures, coordination risks becoming overly expansive and unfocused.

Consistent with previous research, this study reaffirms that the need for coordination arises from the complexity of public health issues, issues that lack simple solutions or clearly defined roles and responsibilities (Weber and Khademian 2008). The involvement of numerous stakeholders with diverse perspectives (Axelsson and Axelsson 2006) further underscores the importance of coordination in ensuring that these issues are not only placed on the agenda but remain there. Without someone explicitly responsible for driving these efforts, they risk being deprioritized in favor of tasks more closely aligned with core organizational missions.

In Sweden, these challenges are amplified by a decentralised public health system, where municipalities have significant autonomy in shaping local health initiatives. The variation in how coordinator roles are structured, from full-time positions to fragmented responsibilities, reflects the difficulty of translating national public health goals into local action. This decentralization, while enabling responsiveness to local needs, contributes to the wickedness of public health coordination: the lack of uniformity, shifting mandates, and diverse stakeholder landscapes, which makes it difficult to establish consistent practices and shared understandings. The Swedish Public Health Agency’s emphasis on coordination across sectors and levels of governance underscores the need for coordinators who can navigate this complexity. In this light, the coordinator role is not only a response to administrative fragmentation but also a strategic mechanism for addressing interdependent, evolving, and contested nature of public health issues in Sweden.

Ultimately, the findings suggest that good coordination is not about narrow efficiency or goal fulfilment. Rather, it is about bringing together the right stakeholders, implementing purposeful activities, and acquiring and sharing knowledge in a way that aligns with policy goals. In line with previous findings (van Rinsum *et al*. 2017, Andersson *et al*. 2019, Eriksson *et al*. 2020), this study shows that coordination is itself a prerequisite for effective collaboration. It enables actors to build a shared knowledge base, develop competencies, and adopt a strategic, long-term, and goal-oriented approach to joint public health practice.

### Methodological reflections

The participants in this study were strategically selected by the Swedish Public Health Agency to ensure that they had experience working with public health in a local context, specifically within the areas of substance abuse, mental health, and suicide prevention. This selection aimed to ensure that participants could share practical insights into coordination. Of the 40 coordinators contacted, 7 declined to participate due to lack of time or experience in the role, while 12 did not respond. Although the reasons for non-response are unknown, it is likely, based on the study’s findings, that time constraints and competing work priorities played a significant role. Those who did participate expressed strong interest in the topic and were eager to share their experiences. The study could further be strengthened by incorporating perspectives from additional stakeholders such as government officials, and frontline personnel responsible for implementing the coordinators’ plans. However, including these perspectives was beyond the scope of the present study.

A key strength of the study was the digital dialogue meeting held after the preliminary analysis. This session allowed for validation of interpretations, correction of any misunderstandings, and further refinement of the results. Participants expressed recognition of, and agreement with, the findings, which strengthens the credibility of the study. From an ethical perspective, the dialogue meeting also enhanced participant involvement, offering them direct feedback and demonstrating that their contributions were valued and used as promised.

## CONCLUSION

By investigating the role of public health coordinators in Sweden, this study aimed to explore how public health coordinators in Sweden work and what their role entails in practice. The findings show that coordination in public health practice involves driving targeted efforts, planning and implementing purposeful activities, engaging relevant stakeholders, and acquiring and sharing knowledge. For this work to be effective, several strategic, operational, and individual prerequisites must be in place. Specifically, to facilitate public health coordination at local levels, efforts should ensure that:

- Local goals for public health practice are established. These goals should be politically anchored and formalised through action plans, steering documents, agreements, and mission statements. This not only legitimises the work but provides coordinators with a mandate to act.
- Coordinators possess knowledge of the municipality’s structures, functions, and roles, enabling them to plan and implement activities and identify and involve the right stakeholders.
- Coordinators have sufficient time, autonomy, resources, and mandate to keep public health issues on the agenda. This requires trusting and supportive leadership, particularly in helping set priorities and manage boundaries.

This study should be seen as a first step in an ongoing effort to better understand the importance of coordination in public health practice. Our focus has been to identify commonalities in coordinators’ descriptions understand how public health coordinators in Sweden work and what their role entails in practice. Future research should explore whether effective coordination differs across public health domains and how conditions and prerequisites vary at local levels. Although evaluating the effects of coordination is inherently challenging, it would be valuable to investigate whether, and how, coordination contributes to achieving the policy goals of public health practice.

## Data Availability

Anonymised data that support the findings are available upon reasonable request from the corresponding author (I.N.G.).
